# Utilization of Antagonistic Interactions Between Micronutrients and Cadmium (Cd) to Alleviate Cd Toxicity and Accumulation in Crops

**DOI:** 10.3390/plants14050707

**Published:** 2025-02-26

**Authors:** Muhammad Shahzad, Ayesha Bibi, Ameer Khan, Ali Shahzad, Zhengyuan Xu, Tagarika Munyaradzi Maruza, Guoping Zhang

**Affiliations:** 1 Department of Agronomy, College of Agriculture and Biotechnology, Zhejiang University, Zijingang Campus, Hangzhou 310029, China; shahzadmuhammad@zju.edu.cn (M.S.); ameer_khan11@outlook.com (A.K.); 11816026@zju.edu.cn (Z.X.); tag.maruza@gmail.com (T.M.M.); 2 Zhejiang Provincial Key Laboratory of Crop Genetic Resources, College of Agriculture and Biotechnology, Zhejiang University, Hangzhou 310058, China; 3 Department of Botany, University of Agriculture Faisalabad, Faisalabad 38000, Pakistan; ayeshabibiuaf@gmail.com; 4 Sanya Nanfan Research Institute, Hainan University, Sanya 572025, China; ali.thathyala3212@outlook.com; 5 College of Tropical Crops, Hainan University, Haikou 570288, China; 6 Zhongyuan Institute, Zhejiang University, Zhengzhou 450000, China

**Keywords:** cadmium, genetic improvement, ion interaction, manganese, transporter, zinc

## Abstract

The presence of cadmium (Cd) in agricultural soils poses a serious risk to crop growth and food safety. Cadmium uptake and transport in plants occur through the various transporters of nutrient ions that have similar physical and chemical properties to Cd, indicating that the genetic manipulation of these transporters and agronomic improvement in the Cd-antagonistic nutrients could be a good approach for reducing Cd uptake and accumulation in crops. In this review, we discuss the interactions between Cd and some micronutrients, including zinc (Zn) and manganese (Mn), focusing on their influence on the expression of genes encoding Cd-related transporters, including *ZIP7*, *NRAMP3*, and *NRAMP4*. Genetic improvements in enhancing the specificity and efficiency of transporters and agronomic improvements in optimizing micronutrient nutrition can inhibit the Cd uptake and transport by these transporters. This comprehensive review provides a deep insight into genetic and agronomic improvement for fighting against Cd contamination and enhancing sustainable agricultural production.

## 1. Introduction

**Figure 1:**
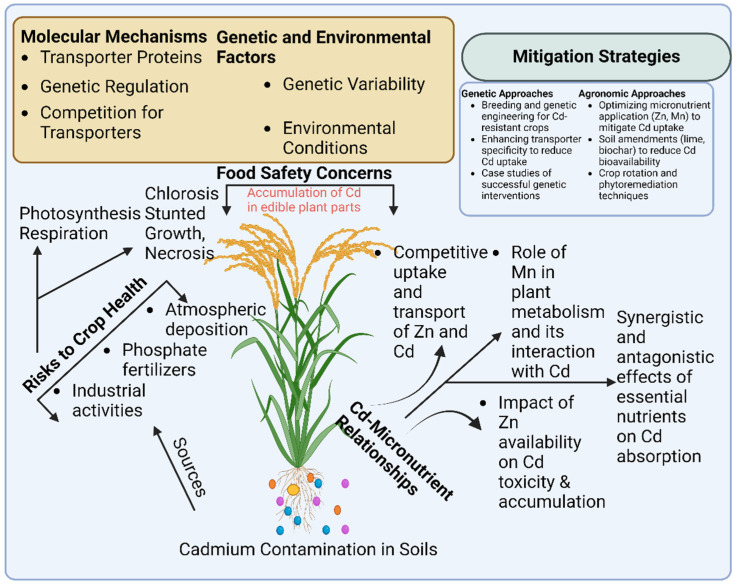
Impact of cadmium uptake and translocation on crop health; molecular mechanisms and mitigation strategies. Different colors including the orange, blue and purple represent Cd, Zn and Mn, respectively.

Cadmium (Cd) is a non-essential metal with no biological function and which is toxic to plants even at low concentrations [1]. Cadmium toxicity severely impacts plant growth and development by inducing oxidative stress, disrupting photosynthesis, inhibiting enzymatic activities, and impairing nutrient uptake and water balance. These effects ultimately lead to stunted growth, leaf chlorosis, and necrosis, significantly reducing crop yield and quality [2,3]. Cadmium accumulation in crops such as poppy poses a significant risk due to its tendency for high Cd content in seeds, leading to potential health hazards to humans. Studies show that Cd transfer to poppy seeds depends on soil Cd levels, plant uptake efficiency, and environmental factors [4,5,6]. Moreover, Cd contamination in agricultural soils bring a huge threat to human health via the food chain because Cd can induce several illnesses, including cancer, heart disease, vascular issues, kidney and liver damage, and disruptions to the male reproductive system [7] (Figure 1).

It is well documented that Cd uptake by roots from soils and transportation from roots to shoots (above ground organs) are dependent on the transporters of some nutrient ions that have similar chemical and physical properties as Cd, such as Zn and Mn [8]. There is a dramatic difference in Cd accumulation among crop species and genotypes within a species [9], which provides the possibility for developing crop cultivars with low Cd accumulation through genetic improvements or gene engineering. The physiological and molecular mechanisms of Cd uptake and transportation in plants have been intensively assessed to explain the distinct differences in Cd accumulation found across various species or genotypes. Cadmium and several micro-elements, including Mn, Zn, Cu, and Fe, interact antagonistically and synergistically in their plant uptake and transportation (Figure 1). Understanding the relationship between Cd and nutrient ions may offer evidence to explain the nature of Cd accumulation in crops [10].

Cadmium contamination has become a serious factor affecting sustainable crop production and human health via the food chain. Hence, it is necessary to make major efforts to control Cd contamination in soil by cutting off the entrance of Cd into agricultural ecosystems, the phytoremediation of Cd-contaminated soil, and reducing Cd bio-availability in soil. Meanwhile, it is more important to develop crop cultivars with high tolerance and low accumulation of Cd. In the past 20 years, numerous studies have examined the physiological and molecular mechanisms of Cd accumulation and detoxification in plants using genomics, transcriptomics, proteomics, and metabolomics, with an emphasis on identifying the genes responsible for Cd uptake, translocation, sequestration, and tolerance in plant tissues [11]. On the other hand, the plants with high tolerance and Cd accumulation can be used in the phytoremediation of Cd contaminated soil. Naturally, certain plant species can accumulate a significant amount of Cd without experiencing any toxicity. It is well known that these plants have special tolerance mechanisms, such as compartmentalization, maintaining redox homeostasis, reducing rhizosphere Cd activity, and transferring Cd to aboveground plant parts [12].

This review explores the complex interactions between cadmium (Cd) and essential micronutrients in plants, focusing on Cd uptake, transport, and detoxification mechanisms. By highlighting the roles of key transporters like *OsZIP7*, ATPase2, *AtNRAMP3*, and *AtNRAMP4*, this review provides insights into how Cd interacts antagonistically or synergistically with nutrients such as zinc (Zn), manganese (Mn), copper (Cu), and iron (Fe). Additionally, it examines the genetic and agronomic strategies for reducing Cd accumulation in crops, emphasizing the potential for molecular and biochemical interventions to enhance crop tolerance to Cd toxicity.

## 2. Cd Uptake and Transport in Plants

### 2.1. Mechanisms of Cd Uptake by Roots

**Figure 2:**
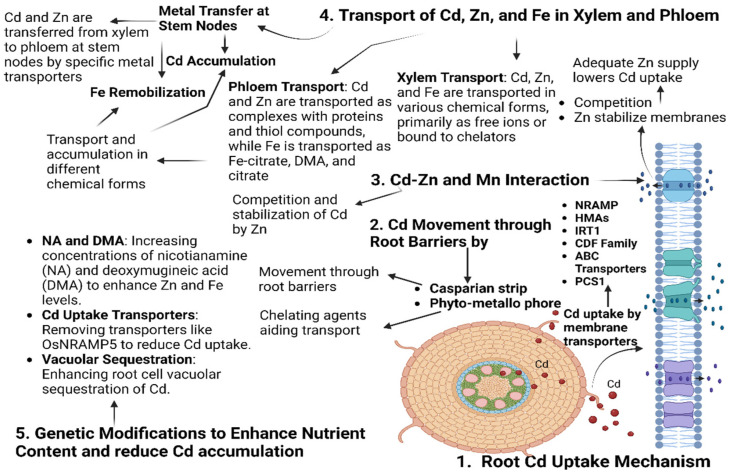
Interactions between cadmium and essential micronutrients in plants: their impacts on metabolic processes and resistance mechanisms.

Cadmium (Cd) in soil is generally insoluble and is not easily absorbed by plants. However, its bioavailability significantly increases when soil pH decreases, meaning that soil acidification can enhance Cd contamination. Plants can affect the bioavailability of cadmium (Cd) by releasing root exudates that alter the rhizosphere’s pH, which can strengthen Cd uptake. In addition, passive diffusion occurs for Cd entering plants through the apoplastic pathway, while the symplastic pathway, an active transport process, relies on electrochemical potential gradients and concentration differences across the plasma membrane [13,14]. For Cd absorbed by plant roots, it must be available for uptake, which is contingent upon the species of the plants, the physicochemical conditions of the soil, and the speciation of the metals [15]. This metal is readily taken up by and delivered to the aerial portions of the plants [16]. The transfer of cadmium (Cd) from soil to grains is a complex and multi-stage process involving the following sequential mechanisms: initial uptake by roots, sequestration within root vacuoles to mitigate toxicity, subsequent translocation through the vascular system to aerial parts, and ultimate partitioning and accumulation in grains [17] (Figure 2).

### 2.2. Role of Metal Transporters

In plants, metal transporters such as *ZIP*, *NRAMP*, and *HMA* families play pivotal roles in the uptake of essential micronutrients. However, these transporters can also facilitate the entry of non-essential and potentially toxic metals like cadmium (Cd) into root cells [18]. For instance, members of the *ZIP* family, including *OsIRT1* and *OsIRT2* in rice, are primarily responsible for iron and zinc uptake, but can inadvertently transport Cd due to its chemical similarity to these micronutrients. Similarly, *NRAMP* transporters, such as *TcNRAMP3*, are involved in the uptake of divalent cations like Fe^2+^ and Mn^2+^, yet they also permit Cd^2+^ entry into plant roots [19]. The *HMA* family, particularly transporters like *HMA2* and *HMA4*, is crucial for translocating essential metals; however, they can also contribute to Cd movement within the plants. This dual functionality underscores a significant challenge in plant nutrition and heavy metal detoxification, as the mechanisms that enable the acquisition of vital nutrients can simultaneously increase Cd accumulation, posing risks to plant health and food safety [20].

### 2.3. Transporter Families Facilitating Cd Uptake and Transport

The transport of cadmium in rice (*OsNramp5*) and barley (*HvNramp5*) is mediated by *Nramp5* homologs, which are also present in wheat (*TaNramp5A*, *TaNramp5D*) and maize (*ZmNramp5*). *Nramp5*, a Natural Resistance-associated Macrophage Protein family member, transports manganese and cadmium in plants [21,22,23]. *OsNramp5* is localized at the distal edges of the exodermis in rice roots, facilitating Cd uptake, which is then either sequestered in vacuoles by *OsHMA3* or transported to the shoots via the xylem. Cd competes with other divalent cations (Ca^2+^, Fe^2+^, Mg^2+^, Cu^2+^, and Zn^2+^) for transport through root cell membranes, and its absorption varies significantly across plant species and genotypes due to morphological and physiological differences [24,25,26,27]. Cd can enter plant roots from the soil solution through cell walls via passive transport. Additionally, active transport mechanisms involve nonspecific membrane transport proteins, such as iron transporters (*IRT*), zinc transporters (*ZIP*), and metal-pumping ATPases, which facilitate Cd movement across the plasma membrane of root cells. Other transporter families, including *NRAMP*, P-type ATPase, *ABC* transporters, *CAX*, *LCT*, and CE, have also been associated with Cd translocation within plants [28,29]. *ZNT1* may be involved in the transport of Cd in the low-Cd accumulation ecotype. At the same time, a high-affinity Cd transporter may also play a role in the transportation of Cd in the high-accumulation ecotype.

### 2.4. Factors Influencing Cadmium Uptake in Plants

Cadmium (Cd) uptake in plants is intricately influenced by micronutrient nutrition, interactions with other metals, and the genetic and molecular machinery of plants. Optimal Zn levels in the growth medium can reduce Cd uptake by competing for transport pathways, whereas Fe influences Cd bioavailability through root–surface interactions and Fe-dependent transporters. However, excessive Zn or Fe may disrupt homeostasis and lead to nutrient imbalances [30,31]. Although Zn supplementation has been reported to reduce Cd uptake in many plant species, contradictory effects have also been reported, depending on soil properties, plant genotypes, and Zn-Cd interactions. Although it is commonly found that Zn can compete with Cd at root uptake sites, reducing Cd accumulation, there are also reports that showed that excess Zn enhanced Cd translocation in plants under certain conditions [32] (Figure 2). Additionally, interactions with other metals, such as calcium (Ca) and magnesium (Mg), can alter membrane permeability and ion exchange, further influencing Cd transport [33]. Plant-specific genetic factors, including the diversity of metal transporter families like *NRAMPs* and *HMA*, regulate Cd uptake and sequestration in vacuoles or cell walls [34]. At the molecular level, transcription factors such as *bZIP* and *MYB* are critical in modulating gene expression for metal homeostasis, leading to variability in Cd accumulation among species and cultivars [35]. Understanding this genetic and molecular diversity is crucial for breeding or engineering plants with reduced Cd uptake, ensuring safer agricultural production and mitigating heavy metal contamination in crops.

## 3. Genetic Regulation and Micronutrient–Cadmium Interactions

### 3.1. Genetic Regulation of Cd Uptake

Rice varieties display significantly genetic heterogeneity in Cd accumulation, offering a valuable resource for identifying functional alleles in improving Cd tolerance [36]. Cd enters vacuoles for sequestration by a Cd transporter that is encoded by *OsHMA3* [37]. The molecular mechanisms of toxic metal Cd in its transport and accumulation in plant tissues have been extensively studied in recent years, and a use for the massive amount of data has been offered by bio-informatic techniques, which have been applied extensively in a variety of plant species [38]. These studies have shown the five groups into which the differentially expressed genes can be categorized, i.e., transporters, organic acids, metabolic pathways, phytohormones, and ROS generation [39]. Transcriptome sequencing revealed that high Cd accumulation in *Nicotiana tabacum* leaves is driven by the coordinated mechanisms, including reduced cell wall binding, weakened Casparian strip barriers, and enhanced xylem loading. Similarly, in barley, genes regulating ion transport, stress response, cell wall dynamics, and reactive oxygen species metabolism play crucial roles in Cd transport and tolerance [40]. In addition, phytometallophores facilitate the transport of metals like Cd by binding to them during their passage through root cells, enabling efficient metal translocation [41]. In short, the genetic regulation of cadmium (Cd) uptake in plants involves a complex interplay of transporter genes, transcription factors, and quantitative trait loci (QTLs) that influence Cd absorption, translocation, and accumulation [42]. Up to date, *Nramp1*, *HMA2*, and *IRT1* have been recognized as the key genes associated with Cd uptake and transport in rice [42].

### 3.2. Micronutrient-Cadmium Interaction in Their Uptake and Transport

Cadmium (Cd) enters plant cells via transporters typically involved in the uptake of essential micronutrients [29]. The uptake of Cd in plants is influenced by several essential elements such as calcium (Ca), copper (Cu), iron (Fe), zinc (Zn), and manganese (Mn) in the rhizosphere solution [43]. Cadmium poisoning often manifests as leaf chlorosis, resembling iron deficiency, as Cd affects the iron accumulation that is essential for chlorophyll synthesis. It is shown that Cd stress reduces Fe uptake and its movement from roots to shoots. In *Arabidopsis*, increased iron availability reduces Cd uptake, whereas in peanuts, an iron deficit enhances Cd accumulation [44]. In graminaceous plants, Fe uptake is mediated by phytosiderophores (e.g., mugineic acids), which can also chelate Cd. The presence of Fe can reduce the availability of phytosiderophores for Cd chelation, thereby limiting Cd uptake. Based on the ability of phytosiderophores to chelate other heavy metals besides iron (Fe), phytosiderophores were suggested to prevent graminaceous plants from cadmium (Cd) toxicity [45,46]. Mn has been found to reduce Cd uptake, as the two metals share similar absorption and transport pathways. Exposed to Mn addition, plants exhibited increased antioxidant enzyme activity, high leaf Mn content, and improved photosynthetic efficiency, leading to the alleviation of Cd toxicity, oxidative stress, and lipid peroxidation [11,47,48]. Zinc (Zn) and Cd interact in several ways due to their similar atomic structures, which complicates efforts to selectively reduce Cd uptake while maintaining Zn transport. Zinc inhibits Cd uptake through shared transporters on the root plasma membrane, and it can mitigate oxidative stress and enhance plant growth [49]. Some studies suggest genotype-specific differences in Cd translocation, and Zn may help reduce Cd bioaccumulation in wheat [50]. Additionally, Zn Transporter7 (*OsZIP7*) and heavy metal ATPase2 (*OsHMA2*) are involved in the xylem loading of Zn and Cu in roots [51] (Figure 2).

Taken together, the interactions between essential nutrients and Cd uptake highlight the complexity of managing Cd toxicity in plants. Optimizing the balance of these elements can help mitigate Cd’s harmful effects, especially in crops like rice, wheat, and peanuts. Table 1 and Table 2 summarize the details of genes and their corresponding functions with identified crops.

## 4. Approaches for Alleviating Cd Toxicity and Accumulation in Crops

Genetic factors control cadmium toxicity and accumulation in crops, but environmental conditions, including soil properties, pH, organic matter, microbial activity, and agronomic practices also have the significant influence on Cd uptake and accumulation. Understanding the interplay between these factors is crucial for developing effective mitigation strategies [63]. Hence, approaches of alleviating Cd toxicity and accumulation in crops are multifaceted, involving genetic improvements for enhancing tolerance and reducing the uptake and transport of Cd, soil remediation for reducing Cd content or bio-availability, and improvements in agronomic management, including irrigation and fertilization, for reducing Cd uptake.

### 4.1. Phytoremediation

An effective strategy for reducing Cd content in soil is bioremediation, a process that leverages the natural capabilities of plants, animals, and microorganisms to restore contaminated environments. Currently, phytoremediation of the contaminated soils by heavy metals, including Cd, has been particularly highlighted and widely used because of its multiple advantages, such as low cost, no secondary contamination, and environmental friendliness [64,65]. The application of Cd phytoremediation is mainly dependent on the availability of Cd hyperaccumulators, the plants with high Cd tolerance, and the root-to-shoot translocation of Cd. For instance, *Noccaea caerulescens* is an extremophile heavy metal hyperaccumulator with a high capacity of Cd and Zn accumulation in shoots. It may be able to effectively phytoextract Cd from soils contaminated with Cd [66]. One factor contributing to Cd tolerance is the increased capacity of rhizosphere microorganisms to generate organic acid to chelate Cd^2+^ [67].

The effectiveness of Cd phytoremediation is significantly influenced by the balance and availability of essential soil micronutrients. Elements like Fe, Zn, and Mn affect Cd absorption by competing for uptake sites or modifying the bioavailability of metals [35,65]. Factors such as soil pH, organic matter content, and microbial interactions further regulate the accessibility of these nutrients and Cd. Plants capable of efficiently absorbing and utilizing micronutrients tend to demonstrate higher tolerance and accumulation of Cd. Enhancing the levels and interactions of soil micronutrients is key for improving the success of phytoremediation efforts [13,68]. Phytoremediation is a promising eco-friendly strategy for Cd-contaminated soil, but it faces several limitations that hinder its widespread application. Firstly, it is time inefficiency and slow remediation rates. Phytoremediation often requires multiple growing seasons to achieve significant Cd removal. Secondly, it is limited by the reasonable hyper-accumulating plants. Commonly high-biomass plants may accumulate less Cd, while hyper-accumulators often have lower biomass. Thirdly, it meets ecological and practical challenges, including the disposal of plant biomass containing accumulated Cd. Improper disposal can cause secondary the contamination of soil and water. Harvested biomass may require controlled incineration, metal extraction, or phytostabilization before safe disposal, thus leading to high costs [69,70].

### 4.2. Genetic Improvement

Genetic improvement represents a promising approach for mitigating cadmium (Cd) stress and toxicity in crop plants, addressing this critical issue from multiple angles. Advances in plant genetics allow for the development of cultivars with enhanced tolerance to Cd, achieved using various strategies such as the manipulation of Cd uptake and translocation pathways, increased sequestration and detoxification mechanisms, and enhanced repair and stress response systems. Key genetic modifications include the alteration of genes involved in Cd transport and binding, as well as the integration of traits that boost the plant’s ability to handle and detoxify heavy metals. Such improvements not only reduce Cd accumulation in edible plant parts, but also enhance overall plant health and yield under contaminated conditions. These genetic strategies are crucial for developing crops that can thrive in Cd-contaminated soils, ensuring food safety and sustainability in agriculture [71,72].

In view of the fact that Cd enters roots and transports from roots to shoots via the transporters of some nutrient ions which have the similar physical and chemical properties with Cd, regulating the expression of the genes encoding these ion transporters might be efficient to control Cd uptake and transport. Zinc (Zn), a crucial microelement for various plant enzymes, competes with Cd for binding sites on root surfaces and in soil, influencing Cd uptake in plants. Zinc-specific transporters, such as *ZIP* (zinc/iron-regulated transporter-like proteins), may inadvertently facilitate Cd entry into root cells and its subsequent redistribution throughout the plant. These Zn transporters, along with others from the *IRT1*, *HMA2*, *HMA3*, *ZIP*, and *NRAMP* families, play a key role in the uptake and translocation of Zn, Cd, and other ions [73]. Modulating metal transporters provides an effective strategy to reduce Cd accumulation while preserving essential nutrient uptake. Silencing *IRT1*, which facilitates both Fe and Cd uptake, can limit Cd entry, but requires careful regulation to avoid disrupting Fe homeostasis [74,75,76]. Overexpression of *HMA3* enhances Cd sequestration into vacuoles, reducing its translocation to edible plant parts. These approaches balance nutrient acquisition with minimized Cd accumulation, offering the potential for safer crop development and improved phytoremediation [77,78,79].

There are multiple steps, from soil to grain, and a number of transporters involved. Thus, it is thought that one efficient way to lessen the amount of Cd that crops absorb is to breed low-grain cultivars that accumulate less Cd. This can be achieved through the use of transgenic technology. The main advantage of transgenic technology is the ability to efficiently express target genes in plants, giving them the correct genetic and biological traits, all without affecting crop quality or yield. For instance, low-Cd rice that accumulates less Cd can be produced without yield penalty by utilizing the CRISPR/Cas9 system to delete *OsNramp5* [80]. While genetic engineering offers promising solutions for developing Cd-tolerant and less-accumulated crops, its adoption is hindered by strict regulatory frameworks, public concerns, and ecological risks. The development and commercialization of genetically modified (GM) crops involve complex approval processes, high research costs, and ethical considerations. Moreover, gene-edited plants might have unintended effects on ecosystem balance, requiring long-term assessment before the widespread implementation of GM crops [81].

### 4.3. Agronomic Practices

A promising and cost-effective method to prevent cadmium (Cd) contamination in food involves using plant nutrients to mitigate Cd toxicity in crops [82]. Plants require a balanced supply of essential nutrients at optimal levels and timings to thrive and reduce Cd’s adverse effects. Farmers often enhance soil fertility to boost crop yields, but effective management of these nutrients is crucial for minimizing soil Cd toxicity. Understanding the interplay between essential plant nutrients and soil Cd is key to achieving this [12]. Essential soil nutrients influence Cd availability and toxicity through both direct and indirect mechanisms [83]. These mechanisms include Cd sequestration in plant tissues, adsorption and precipitation in the soil, competition for membrane transporters, and prevention of Cd accumulation in grains and fruits, all of which work together to reduce Cd solubility and its impact on plant health [84]. Ammonium-based fertilizers (e.g., ammonium sulfate) can lead to soil acidification through nitrification, while phosphate fertilizers release phosphoric acid (H_3_PO_4_), further lowering soil pH, thus increasing Cd solubility in soil and uptake in plants [85,86]. Similarly, potassium fertilizers (e.g., KCl, K_2_SO_4_) also influence soil acidity by promoting cation exchange and leaching, thus affecting Cd availability in soil [87,88]. Fertilizers also determine the speciation of Cd, affecting Cd transport to roots and rhizosphere incorporation [89]. The effectiveness of nutrient application depends on its form and method. Nitrate-based fertilizers are commonly applied as soil amendments, while foliar sprays are effective for micronutrient delivery, improving plant uptake efficiency [90]. The rhizosphere’s nutritional composition, root development, and overall plant growth are all impacted by fertilizer application [48]. Selenium (Se) fertilizers like sodium selenate applied at low concentrations to the contaminated soil can reduce Cd stress and promote plant growth [91,92].

**Figure 3:**
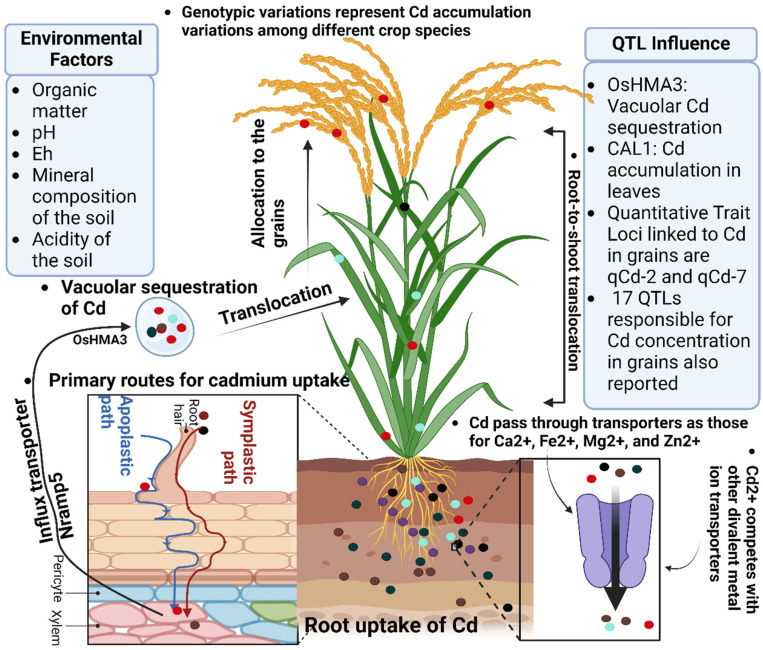
Mechanisms of cadmium uptake and translocation in plants: role of root exudates, transport proteins, and micronutrient interactions. Different colors including the red, green, brown, grape, and black represent Cd, Ca, Fe, Mg and Zn in soil, respectively.

Manganese (Mn) supplementation in the form of MnSO_4_ has been shown to mitigate cadmium (Cd) toxicity in plants by enhancing nutrient homeostasis and reducing Cd uptake. In maize seedlings, exposure to Mn alleviated Cd-induced root growth inhibition, with the degree of improvement correlating with Mn concentration [93,94]. Similarly, in rice seedlings, Mn application reduced Cd uptake and translocation, thereby improving growth and chlorophyll content under Cd stress. These findings suggest that Mn supplementation can enhance plant tolerance to Cd stress by modulating nutrient uptake and distribution [95,96,97,98]. Silicon (Si) can be applied as soil amendments (e.g., silicate minerals), foliar sprays (e.g., potassium silicate), or hydroponic solutions to enhance plant resistance against cadmium stress by improving structural integrity and antioxidant activity [99]. Organosilicon fertilizer also affects Cd accumulation by reducing Cd uptake and accumulation in plants [100]. Meanwhile, silicon fertilizers can increase soil pH, thus reducing the bioavailability of Cd for plant uptake [101] (Figure 3).

The chemical approach to reducing cadmium (Cd) uptake and transport in crops involves the application of foliar sprays or direct soil amendments, which utilize chelation or competition to inhibit Cd absorption. Silicon (Si) and selenium (Se) play a synergistic role in mitigating Cd toxicity by regulating gene expression, immobilizing Cd within root cell walls and organelles, and reducing its translocation to shoots [102]. The use of silicon-rich biochar or Si-fertilizers shows strong potential in further decreasing Cd uptake and movement within plants. Additionally, various other compounds and hormones have been identified as effective in minimizing Cd accumulation, offering a multifaceted strategy to combat Cd contamination in crops [103].

Foliar application of Mn and Zn has been shown to increase their concentrations in plant tissues, thereby potentially reducing Cd translocation to edible parts. Additionally, the interaction between Cd and Zn in the soil can influence their uptake and translocation within plants [25,104]. Applying micronutrient fertilizers, particularly those containing Zn and Mn, can effectively mitigate Cd uptake and accumulation in plants [105,106]. By carefully balancing the application of these micronutrients, it is possible to enhance plant growth and reduce Cd accumulation, thereby improving food safety and crop quality [107].

## 5. Conclusions and Prospects

Cadmium (Cd) contamination poses a significant threat to crop productivity and food safety. While substantial progress has been made in understanding Cd absorption, transport, and resistance mechanisms in rice, critical gaps remain, particularly in the molecular regulation of vacuolar compartmentalization and cell wall sequestration of Cd. Future studies should focus on elucidating the metabolic pathways altered under Cd stress and their interactions with plant nutrition.

Developing crop varieties with reduced Cd accumulation and enhanced tolerance, such as through the manipulation of transporters like *OsNramp5* and *OsHMA3*, is a promising strategy. However, the functional roles of these transporters remain largely unexplored in crops other than rice and Arabidopsis. Bioremediation techniques, including phytoremediation, microbial remediation, and the use of biochar and organic amendments, also offer sustainable approaches for mitigating Cd toxicity in agricultural soils.

Emerging challenges, such as the impact of climate change on soil organic matter breakdown and Cd bioavailability, warrant further investigation. Additionally, identifying novel genes and understanding their roles in Cd uptake, transport, and detoxification will be critical for advancing sustainable agricultural practices and ensuring food safety in Cd-contaminated areas. Comprehensive research and innovative remediation methods are essential to mitigate Cd toxicity and improve crop yields in the face of environmental challenges.

## Figures and Tables

**Table 1:** Key genes involved in cadmium (Cd) uptake and transport, along with their respective protein functions in rice and references.

Gene	Gene ID	Protein	Function	Reference
*OsIRT1*	LOC_Os03g46470	Iron-regulated transporter	Cd uptake	[52]
*OsNramp1*	LOC_Os07g15460	Natural resistance-associated macrophage protein	Cd uptake	[53]
*OsZIP3*	LOC_Os04g52310	Zinc- and iron-regulated transporter	Cd uptake	[54]
*OsHMA2*	LOC_Os06g48720	P-type heavy metal ATPase	Cd transport	[55]
*OsZIP6*	LOC_Os05g07210	Zinc- and iron-regulated transporter	Cd transport	[56]
*OsCCX2*	LOC_Os03g45370	Cation/calcium exchanger	Cd transport	[57]
*OsCLT1*	LOC_Os01g72570	CRT-like transporter	Antioxidation	[58]

**Table 2:** Genes related to cadmium (Cd) transport and their roles across different plant species, including subcellular localization, functions, and references. This table provides insights into Cd uptake, efflux, and translocation in various tissues and species.

Plant Species	Genes	Tissue	Subcellular Location	Function	Reference
*Arabidopsis thaliana*	*AtIRT1*	Roots	Plasma membrane	Cd uptake	[59]
*Oryza sativa* L.	*OsZIP1*	Roots	Endoplasmic reticulum and plasma membrane	Cd efflux	[60]
*Nicotiana tabacum* var. *Xanthi*	*NtZIP4A/B*	Leaves and roots	Plasma membrane	Cd translocation	[61]
*Miscanthus sacchariflorus*	*MsYSL1*	Stems	Plasma membrane	Cd translocation	[62]
